# Planetary Health Education and Capacity Building for Healthcare Professionals in a Global Context: Current Opportunities, Gaps and Future Directions

**DOI:** 10.3390/ijerph191811786

**Published:** 2022-09-18

**Authors:** Muhammad Asaduzzaman, Rifat Ara, Sadia Afrin, James E. Meiring, K. M. Saif-Ur-Rahman

**Affiliations:** 1 Department of Community Medicine and Global Health, Institute of Health and Society, Faculty of Medicine, University of Oslo, 0450 Oslo, Norway; 2 Planetary Health Alliance, Boston, MA 02115, USA; 3 Planetary Health Working Group, Be-Cause Health, Institute of Tropical Medicine, Nationalestraat 155, 2000 Antwerp, Belgium; 4 Infectious Diseases Division, icddr,b, Dhaka 1212, Bangladesh; 5 Health Systems and Population Studies Division, icddr,b, Dhaka 1212, Bangladesh; 6 Department of Infection, Immunity and Cardiovascular Disease, University of Sheffield, Sheffield S10 2JF, UK; 7 College of Medicine, Nursing and Health Sciences, University of Galway, H91 TK33 Galway, Ireland; 8 Evidence Synthesis Ireland and Cochrane Ireland, University of Galway, H91 TK33 Galway, Ireland

**Keywords:** global health, planetary health education, climate change, healthcare professionals, curriculum development, sustainable healthcare education, mini review

## Abstract

The emerging concept of planetary health needs to be discussed in a more organized and sustainable way within the global public health and healthcare disciplines. Therefore, planetary health should be considered a cardinal component of the global academic framework for healthcare professionals. The availability of related curricula and courses is crucial to equip health professionals in this relatively new discipline of planetary health. In this review article, we aimed to explore published articles and online databases of courses to summarize the available planetary health education opportunities and discussions for health professionals, to identify the gaps in resource allocation and to suggest future recommendations. We observed a visible resource inequity in the global south with the lack of a universal planetary health module for healthcare professionals. Additionally, there is minimal inclusion of allied health disciplines in this learning process. We therefore recommend a dedicated network of motivated healthcare professionals and regional hubs with an agenda to ensure a comprehensive, uniform, and inclusive planetary health education curriculum and practice.

## 1. Introduction

Planetary health (PH) has emerged as a new discipline to safeguard our planet in response to climate change, to limit our detrimental interaction with the surrounding ecosystem, and to protect the lives of all with an intergenerational responsibility [1]. However, this multidisciplinary approach requires innovative ideas and impactful solutions to keep the global community on board along with great pledges and courage from policy makers to encounter prevailing concepts [2]. The role of education is central to this aim by developing not only the basic and contemporary understanding of PH, but also by advancing self-reflection towards the wider society and a recognition of the instrumental contributions of others. Therefore, incorporating PH in the education system for all disciplines is a crucial footstep to obtain the change [3]. To be an inclusive and result-oriented holistic model, individual to community-level professional education is much needed, both formal and informal. 

As the name demonstrates, PH has a strong mission to deal with the environmental threats to public health, primarily originating from man-made intervention in the surrounding ecosystem. The Rockefeller Foundation–Lancet Commission on PH [4] has focused on climate events such as ‘ocean acidification, land degradation, water scarcity, overexploitation of fisheries and biodiversity loss’ as grave global health challenges and therefore, has identified healthcare professionals (HCPs) as one of the six key constituencies to solve these issues. PH should be considered as an important notion for HCPs’ teaching and practice-oriented learning agenda [5]. Most importantly, HCPs including clinicians who deal with patients, should be able to educate their clients, for which they need a knowledge base. Recognizing the influence of several relevant action-oriented movements, such as environmental health, eco-health, geo-health, one health and conservation medicine, we strongly argue for a robust foundation for planetary health education addressing all healthcare disciplines involved to make it sustainable and widely acceptable. There is a distinctive modification from other related disciplines mentioned above where HCPs have been given a major implementing role. In this context, HCPs are undoubtedly one of the key actors in achieving PH goals. 

To be equipped with a reliable understanding of PH, HCPs should be informed of the newly adopted PH education framework [6], both as potential learners and effective educators. They need to be aware of the multidisciplinary ‘systems thinking’ [7] approaches such as the intervention and modification of anthropogenic activities affecting the natural ecosystem and public health, climate and social justice and community movements. However, there is dearth of literature to learn about existing PH curricula and learning opportunities for HCPs. In this article, we, as global health researchers and HCPs, have investigated through a scoping review what PH education opportunities (both online and in person courses) are currently available, the existing gaps in resource allocation for regions and allied professions and how we can proceed further to accomplish our role as PH learners and educators through capacity building in the well-being of the planet. 

## 2. Materials and Methods

We searched three bibliographic databases and relevant websites to identify potential articles describing PH education and existing education programs for HCPs. We searched MEDLINE through PubMed, Scopus and Web of Science with the search term “Planetary Health’ AND ‘Education’ for published documents written in English from the year 2000 to 25 April 2022 (Table S1). Rayyan QCRI systematic review software has been used for the screening of the retrieved articles [8]. It is a free web application which is innovated by Qatar Computing Research Institute and can help experts collaborate on the title/abstract and full-text screening phases of the review process. Two review authors independently screened the articles for inclusion. We mentioned the reasons for exclusion of articles during the full-text screening. We used the ‘prioritization and sequential exclusion’ technique at this stage while reporting the reasons for exclusion [9]. Any discrepancies between the independent review authors were resolved through discussion with a third review author. Data were extracted by a single review author and cross-checked by the author team. Disagreements were resolved through team discussion.

PRISMA (Preferred Reporting Items for Systematic Reviews and Meta-Analyses) flow diagram has been used to present an accurate visual summary of the screening process [10]. Articles were mainly selected based on their statement of PH for HCPs, whether it was information about the ongoing or proposed PH curricula or the necessity of PH education in this professional group. Multiple sources of relevant published documents such as original articles, perspective papers, review papers, workshop reports, editorial letters, or comments, irrespective of study types and geographical distribution, were included for this analysis. Data from the selected documents were extracted based on a list of key points, such as publication year, targeted countries, study design, study population, number of graduates, primary outcome, type of education, duration, contents, institutes responsible for the course and language of instruction, and were recorded in a structured excel format. 

In addition to the scientific articles, a manual search to find other PH education resources, which are currently ongoing or soon to be started was performed. We found the Planetary Health Alliance (PHA) as a well-resourced database, containing information regarding education modules related to PH for HCPs such as short and long courses of different categories [11]. We also searched online education platforms such as Future Learn, Coursera and Harvard edX for PH-related educational material. Most of the courses were offered in English with a few in Catalan, Spanish, German and Portuguese. Education programs (both short and long courses) recommended to anyone other than HCPs were excluded from this search. 

## 3. Results

**Figure 1:**
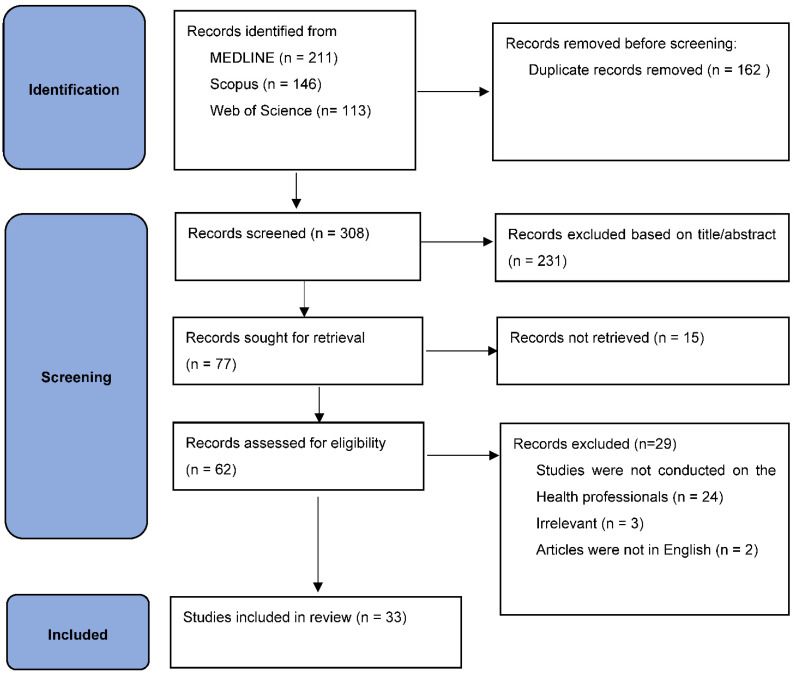
PRISMA flow diagram.

We initially retrieved 470 articles and screened those articles. Following exclusion of the duplicates (*n* = 162), we scrutinized 33 articles from the rest of 308 articles through independent dual screening of the titles and abstracts. Finally, data extraction was considered for these 33 articles after the full-text evaluation (Figure 1).

### 3.1. Current Global Planetary Health Education Schemes for Healthcare Professionals

#### 3.1.1. PH Discussion in Published Literature

Most of the PH education initiatives have been focused on nursing and medical curricula. A summary of 33 articles is presented in Table S2. Most of the included literature was original articles. There were also significant numbers of comments and letters to editors. Moreover, there were two review articles, one case study, one consensus statement, one qualitative study, one report of a workshop and one global survey included in this review. Out of 33 included articles, the ongoing or suggested inclusion of PH for medical curriculum was mentioned in 14 articles [12,13,14,15,16,17,18,19,20,21,22,23,24,25] and nurses were the target group in 6 articles [26,27,28,29,30,31]. Guzmán and colleagues have drafted the overarching PH education framework [3], which needs to be followed by the educators, students, leaders and mentors from all disciplines including the healthcare profession. 

In 2021, the Association for Medical Education in Europe (AMEE) developed an inclusive and globally scalable consensus statement for sustainable health care education, including PH for HCPs [23]. There is another statement known as ‘The American Medical Association (AMA) statement,’ which also emphasizes the education of medical professionals on the public health impacts of global climate change [32]. Focusing on sustainable health care and PH education, several articles have been published to guide on the ‘eco-ethical leadership’ and faculty development in this context [33,34,35]. The sustainable healthcare network in the UK has also shown remarkable progress to include PH modules in their undergraduate and postgraduate medical curriculum [36,37]. Shea et al. [38] assessed the curricula in 160 professional health institutions across the globe (among the members of Global Consortium on Climate and Health Education-GCCHE) including public health, medical and nursing schools. This recent survey reported current climate-related health education in 63% of respondent institutions with 74% institutions considering including more PH courses. However, 71% of organizations experienced several challenges to initiate these courses. We came to know about ‘Medical Students for a Sustainable Future (MS4SF)’, a student-driven, metric-based initiative, first initiated by the Planetary Health Report Card (PHRC) in 2019 at the University of California, San Francisco. This inspiring PH and sustainable healthcare education initiative provides a standardized tool for medical schools to perform needs assessment and track improvement over time. The goal was to make a difference by incorporating PH into medical school curricula, expanding research opportunities, engaging meaningfully with community environmental justice efforts, supporting medical students to organize PH events at the institutional level, and implementing sustainable practices. According to 2022 PHRC report [39], 74 medical schools from the US, England, Canada, and Ireland have included PH education in curricula and assessments. 

So far, the implementation of PH modules in other allied HCPs is limited. Community health workers (CHWs) are globally recognized as the baseline representatives of primary healthcare and community contact points. We could not find any articles addressing their access to PH education except the review paper by Behera et al. [40]. We found a single editorial [41] on the physiotherapy discipline which depicts the moderate progress so far since the adoption of the 2020 environmental physiotherapy agenda [42]. This agenda emphasizes the inclusion of PH curriculum in entry-level physiotherapy education as an important related discipline; PH education is recommended only in a personal viewpoint for the food and nutrition curriculum [43]. Similarly, a single article discussed the importance of PH curriculum for pharmacists, the most accessible allied HCPs in developing countries [44].

#### 3.1.2. Available PH Courses for Healthcare Professionals

Most of the available PH courses are provided through online platforms (Table 1).

The duration of the short courses ranged from 4 h to 5 months, whereas the long courses ranged from 9 months to 6 years. Some of the renowned global online learning platforms, such as the PHE learning lab, Coursera, edX by Harvard, Iversity, Future Learn and TelessaúdeRS, offer massive open online courses (MOOC) in paid and unpaid options available for anyone, but especially recommended for HCPs. CIH LMU Munich and Planetary Health Academy have an online short course called ‘Climate Change and Planetary Health: Initiating and Leading Transformational Change’ produced in German, providing exceptional knowledge on climate change and its consequences. Another German short course, offered by the Institute for Advanced Sustainability Studies, is mainly focused on women’s leadership in PH. Top-ranked institutions such as the Yale School of Public Health, University of California, University of Toronto and University of Michigan have some outstanding online short courses on PH whereas few universities, e.g., Harvard University, University of California and Universitat Pompeu Fabra, Barcelona provide in-person short courses. Three unique short courses related to PH running for 16 weeks are available at the University of Sydney, where HCPs can take this ‘unit of study’ individually or as part of a master’s degree.

Besides these short courses, there are also some other categories, such as project tutorial courses offered by the Humboldt University of Berlin, professional exchange program offered by Alam Sehat Lestari (ASRI) and Health In Harmony. Moreover, the Centre for Sustainable Healthcare often arranges practical virtual workshops for global researchers (Table 1). 

Regarding the long courses, undergraduate, doctoral and postdoctoral programs on PH are few though PH modules are available in many graduate programs globally (Table 2). 

The Dominican University of California offers a ‘Planetary Health Minor’ undergraduate program for one year. Brunel University, London has a BASc program on Global/Planetary health for 3–4 years duration. Only George Mason University offers a PhD program on ‘One Health: A Transdisciplinary Approach’ that includes PH in one semester. The London School of Hygiene and Tropical Medicine and the University of California have postdoctoral fellowship programs for 1 to 2 years that contain PH modules as part of the fellowship. The University of Edinburgh, Open University of Catalonia (UOC) and ISGlobal, University of Exeter, Portland State University School of Public Health, Tokyo Medical and Dental University, University of Global Health Equity, Mel and Enid Zuckerman College of Public Health, The University of Arizona are some major universities that are offering master’s certificate courses for 1–6 years of duration, depending on the part-time/full-time modalities. ISGlobal (Barcelona Institute for Global Health), under the University of Barcelona, provides an online graduate course opportunity of 9 months duration and is conducted in the Catalan/Spanish language. 

## 4. Discussion

Our aim was to understand existing research and practice globally in PH education targeting HCPs. Therefore, we have listed the retrieved literature and online resources based on geographical locations, methods and duration, to discuss our perceived view on this topic. 

We observed initiatives on environmental learning inclusion in the medical curriculum [37] and the publication of the Rockefeller Foundation–Lancet Commission on planetary health [4] in 2015. Many of the published articles summarized the necessity of climate-aware health care providers who require the inclusion of PH modules in the curriculum at both undergraduate and postgraduate levels [13,14,16,19,22,25,38]. The ‘AMEE consensus’ and ‘Sustainable healthcare education (SHE)’ are the most prominent guiding examples in terms of medical PH education [23,36]. A recently published article [45] has focused on the integration of climate related health topics in internal medicine and its subspecialty groups. However, existing postgraduate medical educational frameworks need more knowledge base in the current scenario. 

The first call for action to integrate climate and ecological frameworks in nursing education was published in 2017 [26,28]. The GCCHE (established in 2017), a collaborative platform of medical, public health and nursing schools in the United States, calls for the integrated curriculum synthesis for climate education. Later, an ‘Ecological model for planetary Health’ for nursing curriculum was proposed by McDermott-Levy et al. [29] to address four specific topics—‘Use of Health Care Resources, Air Quality and Extreme Heat, Climate change impact on Mental Health, and Adaptation strategies in Natural Disasters.’

Apart from success stories, there is a noticeable lack of knowledge and resource equity between the Global South and North. The majority of the available PH courses and discussions of curriculum development, presented in this article, have emerged from high-income countries. Similar previous concepts, such as one health, have been developed with an inequitable focus on animal health whilst neglecting the public health and environmental perspective [46]. The emergence of PH as a unique discipline provides an opportunity to develop a truly inclusive educational and research platform. Some have observed the emergence of PH as having a colonial epistemology originating from the global north, which tends to exclude non-human organisms [47]. In disagreement with this statement, we consider PH as a more holistic approach, but the capacity to accommodate all disciplines is not yet optimized. Most of the initiatives for PH education have been developed by institutions and consortia from the global north, illustrating the dominant role of developed countries, mainly from the United States and Europe in this emerging area of education (Table S2). Nevertheless, the current COVID-19 pandemic has induced a global learning environment through online platforms and has reduced the gap to a greater extent, marked in Table 1 and Table 2. The lack of a universal PH module ensuring the basic and uniform planetary knowledge for all types of HCPs is an important concern. There are major gaps in the process of inclusion for patient-oriented allied health disciplines (except doctors and nurses in the global north) such as clinical psychology, speech and language therapy, occupational therapy, physiotherapy, medical assistants, dentistry, community health workers, midwifery and local and indigenous professions (Ayurvedic, Homeopathy, traditional healers). We believe it is important and beneficial to address these professionals with a similar knowledge base on PH. 

Unfortunately, both the curriculum development and modification in medical or allied health professions are time-consuming with institutional and national political bureaucracy [48]. In the global south, colonial herd mentality is manifested in the medical curriculum, e.g., adoption of British curricula in the Indian subcontinent [49]. Additionally, the organizations and education systems that have poor internal integration and little external cohesion, tend to resist new ideas and concept mobilization [50]. A recent survey about the views of physicians and nurses on climate change and health [51] revealed their poor level of knowledge (41%) and unwillingness to engage the public with climate education (31%); 14% even considered public engagement on this issue as professionally or personally risky. Therefore, before approaching curriculum modification, we recommend focusing on faculty development, public awareness and building a dedicated network of motivated physician leaders and regional hubs with a strong political agenda. Additionally, it is also important to involve students and trainees in this effort and to consider developing and implementing curricula on a smaller scale to build faculty engagement.

### 4.1. Limitations

To our knowledge, the current review is the first attempt to summarize the existing PH education literature and course databases for HCPs. There are a few limitations to our approach. Based on our search strategy, we have identified the published literature, which discussed PH curriculum in general for HCPs. However, no specific curricular modules in the context of individual institutions or specialties have been mentioned here. We also categorized the existing PH courses, which are publicly available and found online. However, bibliographic searching or communication with medical schools, consortiums or relevant networks would result in further resources, which could be missed in manual searching.

### 4.2. The Way Forward

With regard to patient-oriented PH management, clinicians play a key role in both prescription and health message dissemination. Considering water and food insecurity, loss of biodiversity and sea level rise due to climate change, Africa, South Asia and Small Island Developing States are the most climate vulnerable regions and will be for many years [52]. Therefore, these regions need to be prioritized in capacity building for climate education as well as in clinical practice due to the high burden of climate related illness. There should be a knowledge base for clinicians in these regions, particularly in planetary well-being [53]. Integrating PH education into so many specialized clinical curricula will be challenging. We argue for a basic course for all general practitioners (GPs) around the globe. However, in service PH training should also be provided to current HCPs in diverse clinical specialties. The ‘Clinicians for Planetary Health’ [54] initiative can lead on this issue in collaboration with the relevant regional and national medical associations, including in the licensing examination. 

## 5. Conclusions

Our study findings highlight the lack of uniform and basic understanding of PH for the global HCP community with inequitable resource allocation and unsustainability in the PH education schemes in developing country settings. Any novel didactic pathway such as PH should be culturally driven considering gender and diversity responsiveness, participatory, and native knowledge and language inclusive, to make it sustainable. The decolonization of PH in terms of both geography and professional disciplines must be warranted. We need to transfer the basic building blocks of PH to all, irrespective of geographic location, resource allocation, race or culture, and for this, we should utilize local language and context. The current medium of most courses in English and less use of local/indigenous knowledge exclude the participation of HCPs from the global south with varying educational levels, such as midwives or CHWs. A comprehensive, and inclusive PH education curriculum should exist with an equal knowledge base for the wider healthcare professional community. 

## Figures and Tables

**Table 1:** Available short courses for planetary health education.

Recommended Courses	Institutions/Platform	Course Type	Duration	Language
Biological Systems and Planetary Health	University of the Philippines Los Baños	Online academic course	14 weeks	English
Health Impacts of Climate Change	Yale School of Public Health	Online certificate program	18 weeks	English
Planet to People’s School for Human and Environmental Health	Teachable/Planet to People	Online short course	1 week	English
Planetary Health: Health of Humanity and the Planet in the Anthropocene	Humboldt University of Berlin	Project tutorial course	180 h	German
Population Health Environment short course	PHE Learning Lab at Pathfinder International and the University of Nairobi	Short course	12 weeks	English
The Health Effects of Climate Change	Harvard University: (edX course)	Online short course	7 weeks	English
Climate Change and Health: From Science to Action Specialization	Coursera, Yale University	Online course	3 months	English
Changements climatiques et santé: prévenir, soigner et s’adapter.	Insitut National de Santé Publique du Québec/Quebec’s National Institute of Public Health	Massive open online course (MOOC)	6 weeks	French
Climate change and health	Iversity	MOOC	6 weeks	English
The Impact of Climate Change on Public Health	Future Learn	Online course	4 weeks	English
Global Health Diplomacy Course	Coursera, Stony Brook University	Online short course	30 h	English
Food, Sustainability and Planetary Health	The University of Sydney	Unit of study course	16 weeks	English
Climate Change and Public Health	The University of Sydney	Unit of study course	16 weeks	English
One Health	The University of Sydney	Unit of study course	16 weeks	English
Rx One Health	University of California	Short course	2 weeks	English
Women’s Leadership in Planetary Health	The Institute for Advanced Sustainability Studies	Online short course	6 weeks to 5 months	German
Green Space and Health	Centre for Sustainable Healthcare	Online workshop	4 weeks	English
Carbon Footprinting for Healthcare	Centre for Sustainable Healthcare	virtual workshop	4 h	English
Planetary Health Exchange Program	Alam Sehat Lestari (ASRI) and Health In Harmony	Professional exchange program	1–3 months	English
Planetary Health and Environmental Epidemiology	Duke Kunshan University	Short course	14 weeks	English
Planetary Health	Dalla Lana School of Public Health, University of Toronto	Online course series	6 weeks	English
Planetary and Global Health Ethics	Dalla Lana School of Public Health, University of Toronto	Online course series	6 weeks	English
Climate Change and Planetary Health: Initiating and Leading Transformational change	CIH LMU Munich and Planetary Health Academy	Online course	90 h	German
Promoting Human and Planetary Health: Tools for a Sustainable Future	Future Learn	Online short course	6 weeks	English
Global Health at the Human Animal Ecosystem Interface	Coursera: The University of Geneva, Institute Pasteur, University of Montreal and Centre Virchow-Villermé/University Paris Descartes	MOOC	8 weeks	English
Act on Climate: Steps to Individual, Community, and Political Action	University of Michigan	Online course	7 weeks	English
An Introduction to Planetary Health	Harvard University	Short course	1 week	English
Salud Planetaria	Universitat Pompeu Fabra Barcelona	Short course	-	Spanish/Catalan
Planetary Health for Primary Care	TelessaúdeRS	Online course	7–8 weeks	English and Portuguese
Planetary Health	TelessaúdeRS-UFRGS and the Planetary Health Alliance	Online course	8 weeks	English

**Table 2:** Available long courses for planetary health education.

Available Courses	Institutions	Type of Course	Duration	Language
Planetary Health Postdoctoral Fellowship	London School of Hygiene and Tropical Medicine	Postdoctoral fellowship program	2 years	English
UCGHI GloCal Health Fellowship	University of California	Postdoctoral fellowship	12 months	English
One Health: A Transdisciplinary Approach	George Mason University	Part of PhD program	1 semester	English
MSc in Global Challenges	University of Edinburgh	MSc certificate course	3–6 years	English
Master’s degree in Planetary Health	Open University of Catalonia (UOC) and ISGlobal	Master’s certificate course	1–2 years	Castellà, Català
MSc Environment and Human Health	University of Exeter	Master’s program	1–3 years	English
Global and Planetary Health Concepts	Portland State University School of Public Health	Graduate course	1 year	English
Master of Public Health in Global Health, Planetary Health curriculum	Tokyo Medical and Dental University	Graduate course	2 years	English
Master of Science in Global Health Delivery	University of Global Health Equity	Graduate course	1 year	English
Master of Planetary Health	ISGlobal Barcelona Institute for Global Health, University of Barcelona	Online graduate course	9 months	Catalan/Spanish
MPH One Health	Mel and Enid Zuckerman College of Public Health, The University of Arizona	Graduate course	2 years	English
Global Challenges (Planetary Health) BASc	Brunel University London	Undergraduate course	3–4 years	English
Planetary Health minor	Dominican University of California	Undergraduate course	1 year	English

## Data Availability

All data are available in the manuscript.

## Supplementary Materials

The following supporting information can be downloaded at: https://www.mdpi.com/article/10.3390/ijerph191811786/s1, Table S1: Search strategies for all databases, Table S2: Summary of the included articles.
