# Supplementary material for: Planetary Health Education and Capacity Building for Healthcare Professionals in a Global Context: Current Opportunities, Gaps and Future Directions
Source: Int J Environ Res Public Health. 2022 Sep 18;19(18):11786. doi: 10.3390/ijerph191811786 (PMC9517386; doi:10.3390/ijerph191811786)
Supplement: Supplementary file 1 [file ijerph-19-11786-s001.zip › ijerph-1888793-supplementary.pdf]

Table S1: Search strategies for all databases:

“Planetary Heath” AND “Education”

Database	Search strategy
MEDLINE	"Planetary health"[All Fields] AND ("educates"[All Fields] OR "education"[MeSH Subheading] OR "education"[All Fields] OR "education"[MeSH Terms] OR "education s"[All Fields] OR "educational"[All Fields] OR "educative"[All Fields] OR "teaching"[MeSH Terms] OR "teaching"[All Fields] OR "educate"[All Fields] OR "educated"[All Fields] OR "educating"[All Fields] OR "educations"[All Fields])
Scopus	TITLE-ABS-KEY(“planetary health” AND education) AND PUBYEAR>2000
Web of Science	“planetary health” AND educat*

Table S2: Summary of the included articles (n=33)

Author	Publication year	Article type/ Study design	Region/Country	Target group/profession	Outcome
Huss et al[1]	2020	Case study	Canada	Medical Students	Beginning with knowledge acquisition and reflection in a university setting, this essay presents a conceived process of education for sustainable health care and planetary health.
England et al[2]	2021	Medical education	Northern Ireland	Medical Students	To assess and contrast the outcomes of a student-driven, metric-based program called Planetary Health Report Card, Planetary Health education curriculum, Interdisciplinary Research, Community Outreach, and Advocacy.
Shaw et al [3]	2021	Consensus statement	Global	Medical students	This Consensus Statement describes the changes that are needed in health professions education, as well as strategies for achieving these changes and a schedule for action that is aligned with the internationally agreed-upon SDGs. It represents the combined vision of health professionals, educators, and students from a variety of health professions, geographical areas, and cultural backgrounds.
Omrani et al[4]	2020	Original Article (Observation al study)	Global	Medical Students	The goal of this study was to survey medical students' impressions of the current status of include climate change, air pollution, and health issues in medical curricula around the world in order to inform advocacy for the curriculum reform that the IFMSA Vision aspires to achieve.
Wicklum et al [5]	2020	Letters: corresponden ce	Canada	Medical students	This study focused on outlining some ways family physicians can advocate for PH in medical education.
Moore et al [6]	2021	Editorial	UK	Medical Students	New way of learning PH.
Ramkumar et al [7]	2021	Original article (Cohort study)	Global	Medical Students	This research focused on a format that allowed for the retention of well-established curricular content while also allowing for the inclusion of other important emerging issues that will have an impact on public health locally and globally, as well as the development of climate-aware health care providers.

Rabin et al [8]	2020	Perspective paper	USA	Medical Students	This opinion post stresses the unique role of medical students in sparking the integration of climate information into the pre-clinical medical school curriculum and suggests subjects for wider curricular integration with the principles stressed in pre-clinical medical education.
McLean et al[9]	2020	Editorial	Australia	Medical Students	Climate change and air pollution have been introduced or integrated into medical curricula around the world, according to the International Federation of Medical Students Association (IFMSA). The essays present worldwide and cross-disciplinary viewpoints on a variety of themes related to the urgent need to educate current and future health professionals to deal with the world's "wicked" challenges.
Gandhi et al[10]	2020	Perspective paper	UK	Medical students	For medical students, sustainability is an essential topic, but postgraduate education has not yet caught up with the increased interest in the subject. Due to logistical restrictions and trainers' unfamiliarity, existing postgraduate educational frameworks are unlikely to provide an effective foundation for incorporating sustainability into teaching.
Slimings et al [11]	2022	Mixed method study	Australia	Medical professionals	A mixed methods approach comprising quantitative mapping of planetary health learning outcomes by measuring pre- and post-intervention. Mapping revealed little integration of environmental issues across the medical program. Student's knowledge score increased by 2.37 points on average; 84.2% of respondents rated the activities as excellent/good.
Lal et al [12]	2022	Mixed method study	Australia and New Zealand	Public health and medicine graduates	Quantitative results showed, epidemiologists were the most common experts involved in design and delivery of this climate-health education curriculum. Qualitative interviews highlighted the ad-hoc role of Indigenous-led content in this field, the barriers of time and resources to develop a coherent curriculum and the important role of high-level champions to drive the inclusion of climate change and planetary health.
Chase et al [13]	2022	Opinion	Global	Doctors and medical students	Health professionals have to learn sustainable healthcare in order to protect planetary health. Education for

					Sustainable Healthcare (ESH) is defined as the process of equipping current and future health professionals with the knowledge, values, confidence, and capacity to provide sustainable healthcare services, including mainstreaming planetary health as an integrated curricular theme.
Herrmann et al [14]	2022	Comment	Global	Medical professionals	All medical associations and organizations who publish medical guidelines should include planetary health in their guidelines. Planetary health should become a mandatory dimension of clinical guideline development.
McDermott-Levy et al [15]	2018	Perspective paper	USA	Nurses	This article identifies teaching initiatives in four climate-related areas: usage of health-care resources, air quality and extreme heat, mental health, and natural disasters throughout undergraduate nursing curriculum.
Schenk et al [16]	2019	Perspective paper	Global	Nurses	This article introduces The WE ACT-PLEASE framework that describes fve content domains of pollution from health care sources. It identifies six key professional elements: Professional Obligation, Leadership, Education, Accountability, Science and Engagement.
Leffers et al[17]	2017	Perspective paper	Global	Nurses	The calls to action, the status of climate change in nursing education, and difficulties and recommendations for nursing education connected to climate change and human health are all examined in this article.
LeClair et al [18]	2020	Scoping review	Global	Nurses	To define the types of strategic measures nurses, take to promote environmental justice (EJ) in peer-reviewed literature through research, education, advocacy, and practice (REAP).
Kurth et al [19]	2017	Review article	USA	Nurses	Nursing and midwifery curricula must keep pace with the changing conditions, evolving science, and higher levels of engagement from populations and patients in planetary health matters. Medical and public health curricula have made shifts to include climate change and health matters. Global Consortium on Climate and Health Education is a collaboration of nursing, public health, and medical school curricula.

Rosa et al[20]	2018	Review article	Global	Nurses	As the needs of the planet evolve, nurses will be expected to further reframe professional literacies from a focus on global nursing toward a paradigm of planetary nursing. To sustain the health and well-being of individuals and populations, nurses must become proponents of sustainability amid planetary unpredictability.
Withers et al [21]	2020	Perspective paper	Global	Global health education	The statement urged the member universities to deploy sustainable PH education in their global health programs.
Walpole et al[22]	2015	Original article (Policy Delphi approach)	UK	Healthcare educators/ Faculty development in clinical settings	This article outlined agreed-upon environmental sustainability learning objectives for medical students. Allowing a diverse group of stakeholders to comment on many drafts of the paper sparked creativity.<br>their involvement in and ownership of the concerns raised the learning objectives that resulted.
Walpole et al[23]	2019	Commentary	UK, Europe and USA	Case studies on clinical settings- Nurses, Midwives, Medical students	To learn more about the learning outcomes and case studies of sustainable healthcare education.
Teherani et al [24]	2020	Original Article (Qualitative study)	USA	Faculty development	The findings of this study can help health professionals' educators create SHE experiences that are relevant, effective, and current, as well as benefit community health.
Tun et al [25]	2020	Perspective paper	USA	Faculty development	Faculty may quickly learn and improve the skills they need to incorporate planetary health and sustainable healthcare into their classes. Faculty development is aided by student-faculty collaboration in co-creating new learning. Integral ethical components of education for sustainable healthcare are engagement with personal views on the environment and advocacy for systemic change.
McKimm et al [26]	2020	Commentary	Global	Faculty development and Health professional leadership	Characteristics of a "eco-ethical" leadership approach to health professions education. The following values and ideas are held, displayed (through day-to-day behaviors and actions), and articulated by eco-ethical leaders.
Guzman et al[27]	2021	Commentary	Global	Educators, Leaders, Mentors	To develop a Planetary Health education framework.

Shea et al [28]	2020	Original article (cross-sectional study)	Global	Health professional institutions	This survey asked whether institutions are considering adding climate-health education to their curricula and if they have encountered challenges in doing so.
Wilkins et al[29]	2020	Perspective paper	Global	Food and nutrition education	The nutrition framework has two goals: public health and environmental health. This approach necessitates the inclusion of competences in food systems, agriculture, and policy in the education and training of practitioners and researchers in the field of food and nutrition education.
Behera et al [30]	2020	Review article	Global	Community health workers	CHWs are critical in introducing the principles of planetary health research and education through planetary health education resources and training curricula.
Maric et al[31]	2021	Editorial	Sweden, Germany, New Zealand, Ireland and USA	Physiotherapists	Because of the complexity of today's social, environmental, and health challenges, we must abandon old professional and academic leadership and competitiveness, build new ways of thinking and doing physiotherapy, and intensify our efforts to communicate and collaborate across all boundaries.
Maric et al [32]	2020	Perspective paper	Global	Physiotherapists	This paper argues that incorporating environmental and sustainability considerations into entry-level physiotherapy education is the single most effective measure we can take as a profession right now to assure the greatest possible contribution to planetary health, both now and in the future.
Maharaj et al [33]	2021	Commentary	Global	Pharmacist	The practice of pharmacy can have an influence on planetary health and therefore practice guidelines and policy that govern the profession must make planetary health a pivot upon which the curriculum is taught, the profession is regulated and quality improvement bench markers are created.

1. Huss N, Ikiugu MN, Hackett F, Sheffield PE, Palipane N, Groome J. Education for sustainable health care: From learning to professional practice. *Medical Teacher* 2020; **42**(10): 1097-101.
2. England V, McPhee H, Wells R, Westwood R. QUB Students: Changing the Climate of Planetary Health Education. *The Ulster Medical Journal* 2021; **90**(3): 142.

3. Shaw E, Walpole S, McLean M, et al. AMEE consensus statement: planetary health and education for sustainable healthcare. *Medical teacher* 2021; **43**(3): 272-86.
4. Omrani OE, Dafallah A, Paniello Castillo B, et al. Envisioning planetary health in every medical curriculum: an international medical student organization's perspective. *Medical Teacher* 2020; **42**(10): 1107-11.
5. Wicklum SC, Svrcek C, Kelly MA. Advocating for planetary health in medical education. *Canadian Family Physician* 2020; **66**(4): 235-8.
6. Moore A. A planetary health curriculum for medicine. British Medical Journal Publishing Group; 2021.
7. Ramkumar J, Rosencranz H, Herzog L. Asthma exacerbation triggered by wildfire: a standardized patient case to integrate climate change into medical curricula. *MedEdPORTAL* 2021; **17**: 11063.
8. Rabin BM, Laney EB, Philipsborn RP. The unique role of medical students in catalyzing climate change education. *Journal of Medical Education and Curricular Development* 2020; **7**: 2382120520957653.
9. McLean M, Gibbs T, McKimm J. Educating for planetary health and environmentally sustainable health care: Responding with urgency. Taylor & Francis; 2020. p. 1082-4.
10. Gandhi V, Al-Hadithy N, Göpfert A, Knight K, van Hove M, Hockey P. Integrating sustainability into postgraduate medical education. *Future Healthcare Journal* 2020; **7**(2): 102.
11. Slimings C, Sisson E, Larson C, Bowles D, Hussain R. Adaptive doctors in Australia: preparing tomorrow's doctors for practice in a world destabilised by declining planetary health. *Environmental Education Research* 2022: 1-16.
12. Lal A, Walsh EI, Wetherell A, Slimings C. Climate change in public health and medical curricula in Australia and New Zealand: A mixed methods study of educator perceptions of barriers and areas for further action. *Environmental Education Research* 2022: 1-18.
13. Chase H, Hampshire K, Tun S. Improving the medical curriculum on planetary health and sustainable healthcare. British Medical Journal Publishing Group; 2022.
14. Herrmann A, Lenzer B, Müller BS, et al. Integrating planetary health into clinical guidelines to sustainably transform health care. *The Lancet Planetary Health* 2022; **6**(3): e184-e5.
15. McDermott-Levy R, Jackman-Murphy KP, Leffers JM, Jordan L. Integrating climate change into nursing curricula. *Nurse Educator* 2019; **44**(1): 43-7.
16. Schenk EC. Environmental stewardship in nursing: Introducing the "WE ACT–PLEASE" framework. *Creative nursing* 2019; **25**(3): 222-31.
17. Leffers J, Levy RM, Nicholas PK, Sweeney CF. Mandate for the nursing profession to address climate change through nursing education. *Journal of Nursing Scholarship* 2017; **49**(6): 679-87.
18. LeClair J, Watts T, Zahner S. Nursing strategies for environmental justice: A scoping review. *Public Health Nursing* 2021; **38**(2): 296-308.
19. Kurth AE. Planetary health and the role of nursing: A call to action. *Journal of Nursing Scholarship* 2017; **49**(6): 598-605.
20. Rosa WE, Dossey BM, Watson J, Beck D-M, Upvall MJ. The United Nations sustainable development goals: the ethic and ethos of holistic nursing. *Journal of Holistic Nursing* 2019; **37**(4): 381-93.
21. Withers M, Cousineau M, Hairi NN, Rampal S, Wipfli H. The Kuala Lumpur Statement on Planetary Health from the Association of Pacific RIM Universities Global Health Program.
22. Walpole SC, Mortimer F, Inman A, Braithwaite I, Thompson T. Exploring emerging learning needs: a UK-wide consultation on environmental sustainability learning objectives for medical education. *International journal of medical education* 2015; **6**: 191.
23. Walpole SC, Barna S, Richardson J, Rother H-A. Sustainable healthcare education: integrating planetary health into clinical education. *The Lancet Planetary Health* 2019; **3**(1): e6-e7.

24. Teherani A, Nikjoo A, den Boer A, Tong MS, Desai A. Community-engaged sustainable health care education. *The Clinical Teacher* 2021; **18**(1): 62-8.
25. Tun S, Wellbery C, Teherani A. Faculty development and partnership with students to integrate sustainable healthcare into health professions education. *Medical Teacher* 2020; **42**(10): 1112-8.
26. McKimm J, McLean M. Rethinking health professions' education leadership: developing 'eco-ethical'leaders for a more sustainable world and future. *Medical Teacher* 2020; **42**(8): 855-60.
27. Guzmán CAF, Aguirre AA, Astle B, et al. A framework to guide planetary health education. *The Lancet Planetary Health* 2021; **5**(5): e253-e5.
28. Shea B, Knowlton K, Shaman J. Assessment of climate-health curricula at international health professions schools. *JAMA network open* 2020; **3**(5): e206609-e.
29. Wilkins J. Nutrition education in the Anthropocene: Toward public and planetary health. *Journal of Agriculture, Food Systems, and Community Development* 2020; **9**(3): 59-69.
30. Behera MR, Behera D, Satpathy SK. Planetary health and the role of community health workers. *Journal of Family Medicine and Primary Care* 2020; **9**(7): 3183.
31. Maric F, Chance-Larsen K, Chevan J, et al. A progress report on planetary health, environmental and sustainability education in physiotherapy–Editorial. Taylor & Francis; 2021. p. 201-2.
32. Maric F, Nicholls D, Mostert K, et al. The environmental physiotherapy agenda 2023. *Oslo (Norway): Environmental Physiotherapy Association (EPA)* 2020.
33. Maharaj S, Moonilal M, Jankie S, Dookeram D. Pharmacist Rethink through a Planetary Health Lens. *INQUIRY: The Journal of Health Care Organization, Provision, and Financing* 2021; **58**: 00469580211020885.
